# Cognitive effects of prolonged continuous human-machine interaction: The case for mental state-based adaptive interfaces

**DOI:** 10.3389/fnrgo.2022.935092

**Published:** 2022-08-26

**Authors:** Marcel F. Hinss, Anke M. Brock, Raphaëlle N. Roy

**Affiliations:** ^1^Institut Supérieur de l'Aéronautique et de l'Espace (ISAE-SUPAERO), Toulouse, France; ^2^Ecole Nationale de l'Aviation Civile (ENAC), Université de Toulouse, Toulouse, France

**Keywords:** adaptive interfaces, mental fatigue, physiological computing, estimation, EEG, eye-tracking, ECG

## Abstract

Operators of complex systems across multiple domains (e.g., aviation, automotive, and nuclear power industry) are required to perform their tasks over prolonged and continuous periods of time. Mental fatigue as well as reduced cognitive flexibility, attention, and situational awareness all result from prolonged continuous use, putting at risk the safety and efficiency of complex operations. Mental state-based adaptive systems may be a solution to this problem. These systems infer the current mental state of an operator based on a selection of metrics ranging from operator independent measures (e.g., weather and time of day), to behavioral (e.g., reaction time and lane deviation) as well as physiological markers (e.g., electroencephalography and cardiac activity). The interaction between operator and system may then be adapted in one of many ways to mitigate any detected degraded cognitive state, thereby ensuring continued safety and efficiency. Depending on the task at hand and its specific problems, possible adaptations -usually based on machine learning estimations- e.g., include modifications of information, presentation modality or stimuli salience, as well as task scheduling. Research on adaptive systems is at the interface of several domains, including neuroergonomics, human factors, and human-computer interaction in an applied and ecological context, necessitating careful consideration of each of the aforementioned aspects. This article provides an overview of some of the key questions and aspects to be considered by researchers for the design of mental state-based adaptive systems, while also promoting their application during prolonged continuous use to pave the way toward safer and more efficient human-machine interaction.

## 1. Introduction

Many domains of study have over the past century seen a rapid increase in efficiency accompanied by a shift to ever more complex systems (Hollnagel, 2009). Operators now work with more complex systems, that are often larger in scale, productivity, and effectiveness, which in turn makes errors much more costly. For instance, less than a 100 years ago, between the world wars, commercial planes usually carried a lot less than 50 people (Linden and Seely, 2011), on the other hand, the worst aviation disaster claimed more than ten times that amount of lives in 1977 (McCreary et al., 1998). There are many examples that highlight the importance of studying the human-machine interaction in order to avoid catastrophic errors. The three Mile Island incident, although not caused by human error was exacerbated by faulty human-machine interaction (HMI). When radioactive hydrogen from a nuclear plant leaked into the environment, operators were inhibited in their efforts to control the damage by a multitude of alarms and faulty interface design (Cummings, 1980). The operators did not regain control immediately, resulting in further leakage. In another accident, a broken light bulb distracted the crew of an aircraft to the point that they did not realize they were flying into the ground (O'Brien and Bull Schaefer, 2020). As these examples show, neither the machine nor the operator is to be seen as an independent unit, and only if both parts perform adequately can safety be ensured (Ke et al., 2018). Other than machines, humans are prone to mental fatigue, resulting in increased risk and errors (Matthews, 2012).

What can be done if the operator cannot just be taken out of the equation by means of automation or by replacement? The effects of Time on Task (TOT), the time since the onset of a task, may be mitigated by adaptive systems. The idea of such systems is to accommodate the mental state of the operator according to their current needs and the current mission requirements (Hansberger, 2015). The ways in which systems accomplish this are vast. Adaptive automation partially or completely automates the operation in given circumstances (e.g., an auto pilot in a plane). Adaptive interfaces, on the other hand, do not automate operations, but change the interaction, by adapting their interface. Examples of this are plentiful, such as adaptive driving modes (Kesting et al., 2007), or automated night and day modes for smartphones (Langley, 1999; Lunn, 2022). These systems are all very useful, yet they are largely operator independent. However, individual differences such as experience and preferences influence how an operator's mental state may change over time (Matthews, 2012). Consequently, adaptive systems need to take into consideration the mental state of the operator. Examples of mental state-based adaptive systems often include a component of physiological computing. The idea of physiological computing is to estimate the current mental state of a human by using physiological measures such as electroencephalography (EEG), or eye-tracking (Roy et al., 2020). Adaptable systems on the other hand avoid these difficulties of estimating the mental state of the operator by leaving the human in control of when and whether to activate any change in automation or interface. For mental state-based system design, adaptive systems are most likely preferable, as they do not rely on the assumption that a human can accurately infer their own mental state (Findlater and McGrenere, 2004).

Surprisingly, relatively little research has explored the possibility of mental state-based adaptive interfaces for operator support during prolonged continuous use. It is the aim of this article to take a step toward that direction, by examining the current state of research within the field to try and answer the following questions:

- What are the effects of prolonged continuous use on cognition and behavior?
- How should an adaptive interface infer the current state of the operator?
- How can adaptive interfaces affect the interaction between humans and systems to mitigate said effects?

This article was meant to address these issues using a non systematic review process. Indeed, the study in this article reviews the effects of prolonged use on cognition as well as research on a mental state based on adaptive interfaces and different classes of metrics for mental fatigue detection. Literature authored in English was selected based on the relevance to the topic and its scientific rigor. As a result of this, the list of effects of mental fatigue on cognition in Section 2, possible and implemented adaptations in Section 3, and the set of metrics for mental fatigue detection in Section 4 is not exhaustive. The principal search engine used for the literature review was Google Scholar. In addition, the ACM (www.acm.org) and IEEE (www.ieee.org) databases were searched. Although no hard inclusion and exclusion criteria were defined, articles that were not peer reviewed were generally avoided. Furthermore, as adaptable systems and HMI are rapidly evolving fields, there was a preference for more recent literature, mostly less than 10 years old. Each of the included articles was assessed based on its scientific rigor. If articles were not deemed rigorous in all aspects they were either excluded or any potential shortcomings in the design were mentioned in the article (Kable et al., 2012).

The following section will examine how prolonged use can affect the HMI by looking at its effects on cognition and behavior.

## 2. Prolonged continuous use, its effects, and its relation to mental fatigue

“Prolonged continuous use” or “prolonged operation," as is defined here refers to the exercising of a task for long periods of time or beyond an expected duration. In experimental sciences, it is often referred to as TOT. Closely related to this concept and almost always studied together is the concept of mental fatigue. Mental fatigue, in contrast to physical fatigue, arises through continuous cognitive effort and may manifest itself in decreased cognitive performance, lack of motivation, or exhaustion (Boksem and Tops, 2008; Ackerman and Kanfer, 2009; Matthews, 2012). This article focuses exclusively on the cognitive aspects of mental fatigue. Mental fatigue is not the only result of prolonged continuous use. Nevertheless, both terms are related (Trejo et al., 2015). Mental fatigue is often studied by having participants perform a task for a prolonged time. Therefore, studies of mental fatigue that make use of this research design inevitably also study TOT. Such a within subjects design is used in Yu et al. (2021), Csathó et al. (2012), Guo et al. (2016), Plukaard et al. (2015), Lorist et al. (2009), Monsell et al. (2003), Krishnan et al. (2014), and Dorrian et al. (2007), while van der Linden et al. (2003a) and van der Linden et al. (2003b), use a different design. In the latter, one group performs a mentally fatiguing, but irrelevant task before performing the main task while another group does nothing before completing only the relevant task.

It is beyond the goal of this review to explore all effects of mental fatigue and TOT in detail. Rather, it will be shown that prolonged continuous use and mental fatigue have effects on critical aspects of HMI. Table 1 summarizes the effects of prolonged continuous use on cognition which are then detailed in the subsequent sections.

**Table 1:** Summary of prolonged continuous use effects on cognition.

**Cognition**	**Effect**	**Duration and reference**
Attention	Decreased top-down attention, no effect on bottom-up attention	35min (Holtzer et al., 2010)
	decreased ACC & Increased RT during visual attention	150 min (Csathó et al., 2012)
	Decreased attention during driving	20 min (Sun et al., 2014)
	Decreased spatial attention	63 min (Guo et al., 2016)
Cognitive Flexibility	Increased task-switching costs (higher RT and lower ACC)	120 min (Yu et al., 2021), 120 min (Lorist et al., 2009), 180 min (Petruo et al., 2018), 60 min (Plukaard et al., 2015)
	Reduced task switching costs if switches were cued	180 min (Petruo et al., 2018), 120 min (Yu et al., 2021), 120 min (Lorist et al., 2009)
	Increased task-switching costs if working memory was involved	120 min (Yu et al., 2021)
	Decreased task-switching costs when given opportunity to prepare before switching	120 min (Lorist et al., 2009)
Situational	Increased preservation	na. (van der Linden et al., 2003a)
Awareness	Decreases planning capabilities	na. (van der Linden et al., 2003a)
	Decreased evaluation of a simulated ATC task	120 min (Krishnan et al., 2014)
	Decreased exploitative behavior	na. (van der Linden et al., 2003b)
	Increased alert fatigue	na. (Blackmon and Gramopadhye, 1995; Bliss et al., 1995; Cash, 2009)

Duration refers to the TOT, if applicable (within subjects designs). RT, Reaction time; ACC, Accuracy.

### 2.1. Mental fatigue and attention

Attention is a critical component in many aspects of human-machine interaction (Roda, 2011). Attention is a broad concept and can encompass many sub types. A simple categorization is accomplished by dividing into exogenous “Top-Down” or endogenous “Bottom-Up” processes (Theeuwes, 1991). Top-Down attention is a voluntary allocation of attention toward some specific feature or object. For example, humans are capable of focusing on just one person speaking, even in a crowded and loud room. This is also known as the cocktail party effect (Bronkhorst, 2000). Bottom-Up attention is the exact opposite, it is involuntary attention that is strongly guided by salient objects in the environment. This is the capability to hear our own name from far away despite not paying any attention to that person (Katsuki and Constantinidis, 2014).

The general consensus seems to exist that mental fatigue has a much more pronounced effect on top-down attentional processes, as demonstrated by Holtzer et al. (2010). In this study, participants had to perform an Attention Networks Test (ANT). The ANT is based on a flanker task (Eriksen and Eriksen, 1974) and interpretation of the behavioral results allows for distinguishing between effects on bottom-up and top-down attention. After performing the task for a prolonged period, bottom-up attention showed no change due to TOT, whereas top-down attention performance significantly decreased. Similarly, Csathó et al. (2012) showed a decrease in performance (increase in reaction time (RT) and decrease in accuracy (ACC) on a visual attention task, as mental fatigue increased. Interestingly, even though participants showed initial performance enhancements, most likely due to learning effects, fatigue in the end resulted in decreased performance. Further support for the effect of fatigue on attention was seen in a study by Sun et al. (2014). Over time, attention decreased and, subsequently, reaction time increased.

Mental fatigue also impacts spacial visual sustained attention. In a study by Guo et al. (2016) participants had to perform a visual task over 63 min in which they had to track a target and respond to random stimuli. The results of this dual-task setup highlighted the participants' inability to maintain fast reaction times and accuracy as mental fatigue increased.

### 2.2. Cognitive flexibility, task-switching, and multitasking

The relationship between fatigue and cognitive flexibility has been less explored, but it may nevertheless be an important variable of success for prolonged use (van der Linden et al., 2003b; Lorist et al., 2005; Plukaard et al., 2015; Yu et al., 2021). Cognitive flexibility is the ability of an individual to rapidly change from one task to another task. This task-switching usually incurs a cost, resulting in increases in reaction time and decreases in accuracy (De Jong, 1995). As complex systems are often composed of multiple sub-systems (see Lim et al., 2018 as an example of the evolution of control surfaces in cockpits), between which operators have to switch, cognitive flexibility becomes of major importance to operations and prolonged continuous use (Squire and Parasuraman, 2010). The term cognitive flexibility is often used interchangeably with task-switching, while some researchers make a distinction between cognitive flexibility, seen as the ability to switch between tasks and task-switching the actual process of switching. For the remainder of this article, we will follow the latter notion (Ionescu, 2012).

Some recent evidence for decreases in task-switching performance as a result of mental fatigue comes from a 2021 study by Yu et al. (2021). In their study, 33 participants had to perform a prolonged task switching experiment. The task consisted of trials where a rule had to be followed, or a new rule had to be adapted. Behavioral results showed fatigue and task-switching effects. Over time, RT and ACC performance decreased. The surprising finding was that over time, the difference between switching trials and regular trials grew larger. Meaning, that beyond impacting general performance, mental fatigue had an even more profound effect on cognitive flexibility. Similar results were reported by Lorist et al. (2009), Petruo et al. (2018), and Plukaard et al. (2015). Petruo et al. (2018) had used a similar paradigm. However, here the task switching was initiated either by an explicit cue or it had to be based on working memory. Results mirrored those in Yu et al. (2021). Furthermore, the study showed that working memory had a particular impact on the task-switching ability when participants were fatigued.

Contrary results have also been shown. Lorist et al. (2000) studied cognitive flexibility, fatigue, and preparation time. The study showed that switch trials resulted in lower performance and that fatigue had an impact on performance. However, an interaction between fatigue and cognitive flexibility was not found. Reaction times during switch-trials were significantly lower if participants had longer inter-stimulus intervals to prepare for the switch. Even though the overall time to perform an action (interval + reaction time) increased, the relative decrease in reaction time suggests, that participants were better able to cope if given time to prepare for a switch. No effects on accuracy were seen, which may be attributed to ceiling effects, as accuracy remained very high at all times. Similar results are reported in Monsell et al. (2003).

While task-switching is strictly sequential, complex systems often require true multitasking. Task-switching and multitasking have often been investigated separately. Task-switching tasks are always tested sequentially, while multitasking (almost always dual-task) involves concurrent task engagement. However, as laid out by Koch et al. (2018), there are many similarities between task switching and multitasking. Many of the characteristics such as switch-costs also exist for multitasking. This has led to a proposal to see task-switching and multitasking not as different entities, but rather as the same process with only the degree to which the task is sequential differing along a continuum (Salvucci et al., 2009). The link between multi-tasking and mental fatigue is to the best of our knowledge not very well explored. Multitasking is more often investigated as a cause of mental fatigue, rather than examining the effect of mental fatigue on multitasking performance (Kudesia et al., 2022). Given that multitasking is argued to exist on the same continuum as task-switching, it is not unreasonable to expect that performance on multitasking may be impacted by fatigue in similar ways as task-switching (Salvucci et al., 2009).

### 2.3. Other effects of mental fatigue and situational awareness

Beyond the main factors that were already explored, it is worth looking at some other aspects that may play a particular role when it comes to interaction with complex systems. The article by van der Linden et al. (2003a) showed that participants' performance on a Wisconsin Card Sorting Test, got significantly worse if they were subjected to a mentally fatiguing exercise beforehand. The Wisconsin Card Sorting Test (Monchi et al., 2001) is a neurological assessment tool, in which participants are presented with cards. They are instructed to sort the cards according to unknown rules that constantly change. Based on feedback, they then have to adapt their behavior. It was shown that fatigued participants took longer to adapt to this changing of rules than non-fatigued participants, a behavior known as preservation. In the same study, participants also completed the Tower of Hanoi task (Rönnlund et al., 2001), testing their ability to plan ahead. Fatigued participants took significantly longer to make their first move, reflecting difficulties with the initial planning. Similar effects were observed following the prolonged operation of an Air-Traffic Control (ATC) task (Krishnan et al., 2014). After performing about 60 min of simulated ATC work, participants took significantly longer for their tasks.

In another study, also relying on a more ecologically valid design, prolonged continuous use was examined in the context of train driving (Dorrian et al., 2007). Professional train conductors had to perform two simulated 8 h shifts of train driving on different days. From no to moderate fatigue, the operators increased the amounts of errors of commission. For example, they used the brakes more than they had to. When extremely fatigued, they no longer made errors of the commission but made a lot more errors of omission. A likely explanation for these errors is due to decreased attention. Errors of omission may also be seen as the failure to explore the environment at hand. Van der Linden et al. investigated this possibility by studying the effect of mental fatigue on systematic exploration behavior (van der Linden et al., 2003b). Participants either had to do mentally fatiguing tasks or wait before completing an experimental computerized task. Within the complex interface, it was observed to what degree the participants used systematic exploration. Mental fatigue proved to have an impact, reducing systematic exploration significantly.

Another difficulty that may arise as a result of prolonged operations is alert fatigue. This phenomenon describes desensitization to safety alerts as a result of repeated exposure to these events (Cash, 2009). This problem has repeatedly been demonstrated in the health care sector but also in mining operations and construction (Blackmon and Gramopadhye, 1995; Bliss et al., 1995). Decreased adaptation to change, difficulties with planning, reduced exploration, errors of omission, and alert fatigue can be seen as independent entities, but may also be seen as parts of the more general concept of situational awareness (SA; Endsley, 1995). SA is a key concept that needs to be considered for any interaction with complex systems. Endsley defines SA as “the perception of the elements in the environment within a volume of time and space, the comprehension of their meaning, and the projection of their status in the near future (Endsley, 1995). Stanton et al. explain, that SA encompasses an understanding of the past and current environment, reflections about potential future events and their meaning/relevance as well as awareness of goals and goal directed behavior (Stanton et al., 2001).

Loss of SA can have dire consequences, as perhaps best highlighted by the gruesome accident of an L1011 Lockheed Tristar of Eastern Air Lines in Florida. The pilot, co-pilot, and flight engineer of flight 401 did not realize they were losing altitude, as all three were focused on repairing a broken light bulb (O'Brien and Bull Schaefer, 2020). SA is simultaneously a vital factor during complex operations and at perpetual risk to be undermined by mental fatigue, making it an attractive target for support through adaptive interfaces, which are explored in the following section.

## 3. Adaptive interfaces and the question of how to adapt

### 3.1. General considerations for adaptive interfaces

Adaptive Interfaces aim at improving human-machine interaction by changing their behavior to accommodate the needs of the users (Hansberger, 2015). Before considering the context of prolonged continuous use, it is worth exploring some of the general considerations that are known to influence the efficacy of adaptive interfaces. Adaptations are not all or nothing phenomena, and careful calibration of several variables is required to improve the performance of operators (Taylor et al., 2013).

#### 3.1.1. Trust

For the interaction between humans and machines trust is of vital importance. If an operator has no trust in a system they may decide not to engage with it, consequently not making any use of its assistance. On the other hand, putting too much trust in a system can have similarly detrimental effects (Lee and See, 2004). For instance, the crew of the Panamanian passenger ship Royal Majestic placed too much trust in their auto navigation systems. Complacency over 24 h resulted in the ship running around, inflicting costs of over $7 million. Fortunately, there were no fatalities (van de Merwe et al., 2016).

To avoid both over-trust and mistrust, Lee and See (2004) propose the notion of calibrated trust for automated systems. Calibrated trust occurs when the trust an operator has in a system is equal to the true trustworthiness of the system. To achieve this, three variables are identified as critical components:

1. Performance refers to how well the automation is performing and has performed.
2. Process is the degree, to which the operator understands, how the automation operates.
3. Purpose finally explains, to what extent the automation performs what it was intended to do.

Negligence in accommodating these dimensions during the development of an adaptive system can result in both mistrust and over-trust. In automation based adaptations, this is easy to imagine, as a low performance system will likely result in the operator denying the automation of tasks.

The need for trust in automation is being continuously explored, while questions regarding trust in adaptive interfaces remain, to the best of our knowledge, unanswered.

Mental state based adaptive interfaces have to make some form of inference on the current mental state of the operator (Aricò et al., 2016; Park et al., 2021). These metrics that are explored extensively in Section 4 determine when and how a system will adapt. The operator has to have trust in the ability of the system to accurately predict their own current mental state. This is further exacerbated when a system decides to trigger a system adaptation. The triggering will inevitably signal the operator about the estimation of the system on the current interaction. Any discrepancy between the operators' perception of the interaction and that of the interface will probe trust between the human and the system. If the operator does not trust in the accuracy of the interface, they might decide to disengage. This applies equally to adaptive automation. To the best of our knowledge, this area of research is not very explored, but given the rise of adaptive systems, exploring trust in adaptive systems may be critical for future advances.

In order to incorporate trust during the design of an interface, several rules should be appreciated. Again following the advice of Lee and See (2004), for adaptive automation, it is proposed to:

1. Design for appropriate trust, not greater trust.
2. Show the past performance of the automation.
3. Show the process and algorithms of the automation by revealing intermediate results in a way that is comprehensible to the operators.
4. Simplify the algorithms and operation of the automation to make it more understandable.
5. Show the purpose of the automation, design basis, and range of applications in a way that relates to the users' goals.
6. Train operators regarding its expected reliability, the mechanisms governing its behavior, and its intended use.
7. Carefully evaluate any anthropomorphization of the automation, such as using speech to create a synthetic conversational partner, to ensure appropriate trust.

#### 3.1.2. Design considerations for effective adaptive interfaces

There are many other aspects beyond trust that need to be considered to achieve high usability for adaptive systems (Hou et al., 2007). Understanding the process, or the inner workings of adaptations, is not only critical for trust building but has been shown to play a role in the overall success of adaptions (Scott et al., 2006; Kiefer et al., 2017; Xing et al., 2021). This claim is supported in the study of Saint-Lot et al. (2020), where understanding the adaptation resulted in significantly better performance. Another important aspect is to allow the operator to be aware at all times of what the adaptation is currently doing (if it is activated or not Pritchett et al., 2014). Kiefer et al. (2017) and Scott et al. (2006) go even further and propose that the operator should have the capability to take control (deactivate the adaptation) at all times. Pritchett et al. (2014) stress the importance of handling interruptions.

The operator may get “out of the loop” either by adaptations being interrupted or by the adaptation presenting an interruption to its current tasks itself. Pritchett et al. (2014) place further emphasis on accommodating individual differences in character, preferences, and skills. This point is also raised by Hou et al. (2011). While designing adaptations secondary effects need to also be taken into account. An adaptation may prove successful on one measure while decreasing performance on another one. As in the study by Chen and Barnes (2014), where behavioral performance increased with adaptation, while Situational Awareness decreased. The problem of secondary effects opens up the more general question of how to evaluate the effectiveness of an adaptation. In simple tasks, this may be relatively easy, but as soon as the tasks become more akin to simulations of realistic complex environments, a multitude of variables need to be accounted for (Pritchett et al., 2014). The evaluation of an adaptive system is a critical aspect to consider when designing and testing a system. Furthermore, a system needs to be designed in a way, that it can function despite the human not doing what they were intended to Pritchett et al. (2014). Boundary conditions also deserve attention, as the system needs to be optimized as to when an adaptation should be activated or deactivated (Pritchett et al., 2014). Scalability and the possibility to increase complexity should also be taken into consideration. Designs that do not allow for this, lack realism and ecological validity (Hou et al., 2011). Adaptive systems, according to this doctrine, should be built in a way that they could accommodate future additions such as multiple levels of interactions, multiple modes, incorporating individual differences, adding a hierarchy of adaptations, and allowing for multiple ways to activate and deactivate the adaptations (Lajos, 1995; Höök, 2000). Finally, operator comfort is another noteworthy consideration when designing interfaces, even more so for prolonged use. Using visually comforting stimuli within an interface may reduce ocular fatigue (Ladouce et al., 2021). These considerations do not necessarily limit the design options regarding adaptive systems, but they also guide toward future, more viable systems.

### 3.2. Adaptive interfaces in the context of prolonged use

**Figure 1:**
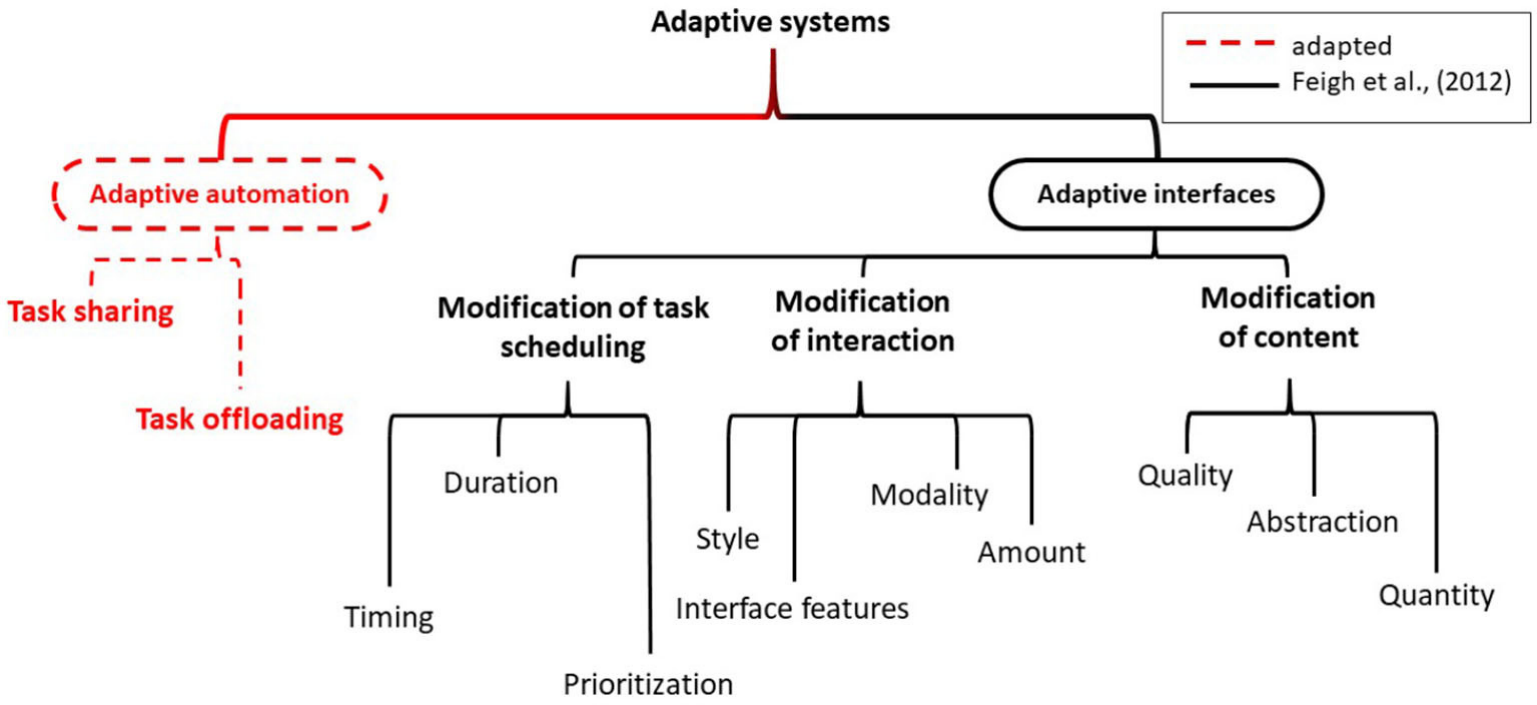
Proposed provisional taxonomy of all types of adaptations based on Feigh et al. (2012).

In their article, Feigh et al. (2012) propose a provisional taxonomy of all types of adaptations. In this taxonomy, no clear distinction between adaptive automation and adaptive interfaces is made. This difference deserves special consideration for prolonged continuous use. Adaptive interfaces in which large changes in the levels of automation occur (Parasuraman et al., 2000), may result in the change in the task being so fundamental, that it can no longer be considered as the same task and, therefore, no longer prolonged continuous use. For this reason, we propose an adapted taxonomy in Figure 1.

Central to the question of how to adapt an interface, is the question of what the goal of the adaptation is. To accommodate the effects of prolonged use and mental fatigue may mean inhibiting their onset. Alternatively, adaptive systems may set out to mitigate the effects of prolonged use, and accommodate the changes in cognition of the operator. Some research has shown the effects, of caffeine, stream-bathing, chicken essence, and physical activity as ways of reducing mental fatigue (Mizuno et al., 2009; Yamano et al., 2013; Van Cutsem et al., 2018; Jacquet et al., 2021), but these options lack in practical applicability in many critical situations. External motivation, especially in form of a reward, has on the other hand shown to effectively reduce mental fatigue (Hopstaken et al., 2015). Nonetheless, motivation may not be a viable solution due to two issues: It is unclear how long the effect of external motivation on an operator lasts and as suggested by Christie and Schrater (2015) it may only allow for short term mitigation. In addition, systems that deliver a reward tend to result in exploitation behavior by operators. The sole objective becomes maximizing direct reward (the external motivation), thereby neglecting other aspects of the system (Dubey, 1986). As direct mitigation of fatigue seems to have very little practicality in this context, efforts to contain the effects of mental fatigue seem more viable. In the following sections, we explore adaptive interfaces and ideas for adaptive interfaces related to operators' attention, cognitive flexibility, and situational awareness. Table 2 summarizes adaptations for different types of mental states, as well as their effects and the application domain.

**Table 2:** Summary of adaptations for various mental states.

**Article**	**Development stage**	**Category of adaptation**	**Adaptation**	**Mental state**	**Effects**	**Domain**
Parasuraman et al. (2009)	Implemented	Automation	Automated target recognition	SA	Improved performance and increased SA	UVC
Aricò et al. (2016)	Implemented	Modification of content	Highlight aircraft and danger	WM	attentional load reduction	Aeronautical
Park et al. (2021)	Implemented	Modification of content	Operator notification about their mental state	AT	Successful recovery of operator AT	UVC
Piechulla et al. (2003)	Implemented	Modification of interaction	Suppression of phone calls	AT & WM	Monitoring traffic environment and variables of driving dynamics	Automotive
Scott et al. (2006)	Implemented	Modification of content	Summary of events after interruptions	SA	Increased SA recovery	UVC
Smallman and John (2003) and John et al. (2005)	Implemented	Modification of content	Changes made explicit	SA	Increased response speed and increased accuracy	Marine
Roldán et al. (2017)	Implemented	Modification of interaction and of content	Relevant UASs selected for manual control and high risk UASs highlighted	SA	Improved performance, and decreased SA (without virtual reality)	UVC
Saint-Lot et al. (2020)	Tested	Modification of content	Screen covered in red hue to alert	AT	Increased detection rate; Understandability increased performance	Aeronautical
Taylor et al. (2013)	Tested	Adaptive Automation abd Modification of content	Auditory alerts supporting visual perception cognition; Driving automation	WM	Diving automation increased WM and disengagement of driver & Auditory alerts reduced workload	UVC
Marquez and Cummings (2008)	Tested	Automation	Automation of task preparation	SA	Improved performance; decreased SA abd automation bias	Aerospace
Arrington and Logan (2004)	Proposed	Task scheduling modification	Allowing for voluntary switches between tasks	CF	Switch costs reduction	Non partial
Lorist et al. (2000)	Proposed	Modification of task scheduling	Task switch cueing	CF	Reduction of switch costs	Non partial
Rubinstein et al. (2001)	Proposed	Modification of task scheduling	Task switch cueing	CF	Reduction of switch costs	Non partial
Petruo et al. (2018)	Proposed	Modification of task scheduling	Reducing working memory during task-switching	CF	Reduction of switch costs	Non partial
Peysakhovich et al. (2018)	Proposed	Interaction modification	Screen removal if looked at for too long	SA	n.a.	Aeronautical

SA, situational awareness; WM, working memory; CF, cognitive flexibility; AT, attention; UVC, unmanned vehicle control. Non partial refers to studies that were not conducted in the context of any specific domain.

#### 3.2.1. Attention

Adaptive interfaces aimed at mitigating problems of attention have generally taken one of two approaches. Most adaptations, be it for unmanned vehicles, robots, etc. have used automation of some tasks to allow the operator to focus on a reduced set of tasks. Parasuraman et al. (2005) examined if partial automation of responsibilities could help operators during unmanned vehicle operations. Participants had to control both a UAS and an unmanned ground vehicle. A performance based system of adaptive automation was integrated into the task. If the performance of operators on a change detection task deteriorated, then parts of their operation were automated. A significant improvement in attention was shown, if operators were supported by the adaptive automation, as opposed to a condition without adaptive automation.

Alternatively, a set of studies have explored exploiting the benefits of bottom-up attention by adapting the salience of alarms and information, thereby decreasing the risk of misses. In order to accommodate increased mental workload in air traffic controllers, Aricò et al. (2016) proposed several adaptations, of which two focused on attention. To facilitate locating an aircraft, the system had the ability to highlight aircraft if they were calling the controller. In addition, if there was a risk of a mid-air collision, the interface would show a specific animation, highlighting the emergency. The result of this study showed, that if adaptations were activated during high mental workload, these were capable of improving performance and allowing for recovery from high mental workload states. A similar adaptive interface was used to increase the attention of UAS operators. In a preliminary study, participants had to fly a simulated UAS, while attention was monitored using a mobile EEG setup. Whenever the participants' attention decreased, they were notified of that decrease with an alert (Park et al., 2021). Effects were not significant, but this might be due to the small sample size (*N* = 4). Success was also seen with the air traffic control “Red Alert” system. Here, the entire screen was draped in a red hue, if there was a risk of a mid-air collision between aircraft. The increase in salience improved the behavior during the ATC task (Saint-Lot et al., 2020). The effectiveness of alarm-based systems is closely related to their accuracy. Inaccurate alarms should be avoided at all costs, as both false alarms and nuisance alarms can be detrimental to performance (alarm fatigue; Onnasch et al., 2014; Wickens and McCarley, 2019; Ruskin et al., 2021). Therefore, several adaptive interfaces have used alarm suppression to increase operational performance. These systems not only avoid alert fatigue but also allow operators to focus attention on relevant objectives, rather than low-priority alarms (Liu et al., 2001; Breznitz, 2013). This has been shown to be effective in chemical plants, where an alarm appeared approximately every 2 min, risking that the operator would disregard a true alarm (Liu et al., 2001). The concept was also applied to car phones, which would automatically direct calls to the mailbox if the mental workload of the driver was too elevated (Piechulla et al., 2003). Comparable to this are Airbus aircraft, which suppress alarms that are not critical during take-off and landing, as focusing on the task at hand may be disturbed by unnecessary alarms (Airbus, 2008). Mirroring the complexity of the concept of attention itself, the examined methods of mitigation differ widely in their approach and may even seem to contradict each other. Adaptive interactions targeting attention may best be summarized, not as an effort of adding or removing alerts and information, but rather as an effort of effective attentional management. The attentional load needs to be carefully calibrated respecting the operational demands, state of a given mission, and state of the operator (Piechulla et al., 2003).

#### 3.2.2. Cognitive flexibility

The effects of task-switching in complex operations are well explored in the literature (Vandierendonck et al., 2010; Koch et al., 2018 for review), however relatively little research has been conducted on adaptive systems and cognitive flexibility. Some studies still point toward some possible solutions for reducing task-switching costs. Arrington and Logan (2004) investigated if voluntary switches, as opposed to scheduled switches, could reduce task-switching costs. Surprisingly, it was shown that task-switching costs still persisted in the form of higher reaction times even if switches were voluntary. Similar effects on accuracy were seen in a follow-up study (Arrington and Yates, 2009). Making switches voluntary seems to reduce the tax on cognitive flexibility, but within complex operations, it is questionable if switches can be made voluntary or have to occur at a specific time or as a reaction to critical incidents. A promising idea is to provide operators with preparation time or cues before switching. As explored in a previous section, some studies (Lorist et al., 2000; Monsell et al., 2003) showed that preparation time and cuing could reduce reaction time in trials (overall reaction time was not shorter if the added preparation time was taken into account). The effect of preparation on accuracy is not conclusively reported in the articles, as error rates were low across all conditions (possible ceiling effect) and no statistics were reported. Rubinstein et al. (2001) reported error rates in their study on cued task-switching. While not reporting any statistics regarding the significance of the data, an almost 30% reduction in errors was seen if trials were cued. These results make cuing task-switches a promising candidate for adaptive interfaces, especially if short delays are acceptable for increases in accuracy. As Petruo et al. (2018) had found, prolonged use in combination with the necessity of using working memory may be particularly detrimental to cognitive flexibility. Adaptive systems that outsource working memory-might prove viable. Interestingly, adaptive automation may have a negative effect on cognitive flexibility, as the act of automating itself may incur switching costs for the participant (Squire et al., 2006; Reinerman-Jones et al., 2011).

#### 3.2.3. Situational awareness

Situational awareness is a critical component in complex operations (Zhang et al., 2021). Several adaptive systems have been tested to improve the situational awareness of operators in different contexts. As with attention and cognitive flexibility, automation is a lot more explored than other forms of adaptation. The results are quite striking, as SA is often negatively impacted by automation (Chen and Barnes, 2014). In one study, participants either had an agent use a path determined by the operator or an automatically proposed path, performance on the objective task measures was better with automation, but participants had a significantly decreased SA (Marquez and Cummings, 2008). In a meta-analysis of the effects of "Level of Automation" (LOA) on task performance, the authors offered a very vivid analogy, stating that LOA can be described by the lumberjack effect: *"the higher they are, the further they fall"* (Onnasch et al., 2013). Their analysis of 14 studies revealed, that Level of Automation had an overall negative impact on performance. Diverting from automation, Scott et al. (2006) proposed an interruption assistance interface. The idea being that an operator of multiple UASs could recover SA after a distraction. Two interfaces were compared to a baseline condition with no aid. Either a short playback of what had recently happened was displayed, or a timeline with bookmarks of events was provided. It was shown that following an interruption, both types of interfaces aided in recovering SA with the bookmark system performing marginally better. A similar tool was developed by John et al. (2005) and Smallman and John (2003). The Change History EXplicit tool was developed to automatically detect changes in a monitoring task and then notify as well as help an operator understand these changes. The implementation resulted in an increases in response speed of 80 and 150% increase in accuracy. An entirely different route was proposed by Roldán et al. (2017). In order to keep SA, they tested an adaptive interface and virtual reality (VR), for the control of multiple UASs. Adapting to the situation, the UAS that had the highest risk indicator would be highlighted and the most relevant UAS would be chosen for manual control. These conditions were either presented within a conventional interface or in an immersive VR simulation. The results proved that VR was an effective way of increasing situational awareness as well as reducing workload. The adaptive part of the system on the other hand decreased situational awareness if it was not coupled with virtual reality. A final adaptive approach to SA was proposed by Peysakhovich et al. (2018). The proposal envisions a system equipped with eye-tracking that detects fixations on objects in the cockpit and temporarily removes said objects if looked at for too long. By doing this, the pilots' attention is re-engaged allowing for recovery of SA.

Adaptations differ widely in their design, goals, and success, nevertheless, the findings may perhaps best be summarized by Onnasch et al. (2014) stating : “*Automation helps when all goes well, but leaving the user out of the loop can be problematic because it leads to considerable performance impairment if the automation suddenly fails.”* As shown, this claim generalizes to other forms of adaptive interfaces and also beyond SA.

## 4. Markers/triggers for adaptation

As outlined by Feigh et al. (2012) successful adaptive interfaces do not only depend on the type of adaptation, but also on the trigger or the metric that determines when an adaptation should be activated/deactivated.

We here propose that metrics that allow for operator mental state estimation can be categorized into being either covariate (1), behavioral (2), or physiological (3). (1) Covariate metrics, are completely independent of the operator. This can range from TOT to the type of mission, time of day, weather, or state of the system. The important aspect is that an adaptive system purely based on covariate metrics would, in the same situation, behave identically independent of any operator. (2) Behavioral metrics then encompass all metrics based on the classical system input devices such as keyboard, computer mouse, and joystick. This includes a behavioral performance that is measurable by the system, such as reaction times or accuracy, but could also extend to conscious input, such as in activating an autopilot (adaptable interfaces). (3) The major difference between behavioral and physiological measures is the need for a specific sensor or specific configuration of a sensor to capture any physiological measure hence creating a new system input device. Typical examples may include electrocardiography, electroencephalography (EEG), and eye-tracking. From these sensors' signals, metrics can be extracted, in both the temporal and the spectral domains (refer to Roy et al., 2020 for a review). Next, a decision has to be made based on the chosen metric(s). If automated, this decision can either be made using a rule-based/heuristic approach, reinforcement learning, or a machine learning approach (Sikander and Anwar, 2019; Todi et al., 2021). Machine learning in this specific context may be divided into traditional machine learning (e.g., linear discriminant analysis and support vector machines Izenman, 2008; Pisner and Schnyer, 2020) or Bayesian optimization. Table 3 lists examples of metrics modulated by prolonged continuous use that may be used in adaptive systems.

**Table 3:** Examples of metrics modulated by prolonged continuous use that can be used for adaptive system design.

**Type**		**Metric**	Effect*	Type of study**	**References**
Covariate	Time	TOT	↑	Operator monitoring	Rodríguez-Fernández et al. (2017)
		Time of day		Operator monitoring	Sikander and Anwar (2019)
Behavioral	Driving	Steering corrections	↓	Operator monitoring	McDonald et al. (2012), Li et al. (2017)
	Computer	Mouse and keyboard		Characterization	Pimenta et al. (2013)
Physiological	EEG	α and θ band power	↑	Multiple	Borghini et al. (2014), Roy et al. (2014), Jahanpour et al. (2020), Tran et al. (2020)
		Left connectivity	↓	Operator monitoring	Sun et al. (2014)
	fNIRS	Connectivity		Operator monitoring	Dehais et al. (2018)
		Frontal oxygenation	↓	Operator monitoring	Ahn et al. (2016)
	Occular activity	Blink Rate and duration	↓	Review	Borghini et al. (2014)
		Saccade Velocity	↓	Review	Di Stasi et al. (2013)
		Fixation and focus	↓	Characterization	Roy et al. (2016a)
	Cardiac activity	Heart rate	↓	Operator monitoring	Lal and Craig (2001), Ahn et al. (2016)
		Heart rate variability	↑	Multiple	Zhao et al. (2012), Borghini et al. (2014), Melo et al. (2017), Huang et al. (2018)
	Body posture			Operator monitoring	Furogori et al. (2005), Ansari et al. (2021)

Upwards arrows indicate an positive correlation with mental fatigue and vice versa.

* If no effect is displayed this is either due to the effect not being explicitly reported or due to the effect being to complex for the table;

** Operator Monitoring includes studies regarding mental fatigue estimation; Characterization refers to studies that experimentally show the effects of mental fatigue, but do not estimate mental fatigue, while Multiple includes combinations of any of the categories.

### 4.1. Covariate and operator independent metrics

Covariate metrics for adaptive interfaces are commonly used. For example, time of day may trigger a smartphone to toggle to night mode (Lunn, 2022), a car may adapt its driving characteristics based on the type of road it is on (Kesting et al., 2007), or, as mentioned above, Airbus aircraft can suppress alarms in certain flight phases (Airbus, 2008). It is not surprising, that adaptive interfaces and adaptive automation for prolonged continuous use rarely rely on covariate metrics alone, as they fully neglect the role of the operator. However, some studies have made use of covariate metrics in combination with other forms of metrics. For example, TOT played a critical role in a time series clustering model that was also informed by the behavior of participants in a simulated UAS task (Rodríguez-Fernández et al., 2017). Similarly, in the automotive domain mathematical models have used time of day to produce models of driver fatigue (Sikander and Anwar, 2019). Covariate metrics being independent of their operator may be seen as a disadvantage, however, it is our opinion that operator independent systems also bring advantages. They do not require calibration on the operator and the system makes far fewer assumptions on the mental state of the operator, therefore reducing the need for trust. Finally, due to their simplicity and non obtrusiveness, they may be relatively practical.

### 4.2. Behavioral metrics

Behavioral metrics offer some unique advantages as they remain non obtrusive and require no additional equipment, while also allowing for increases in accuracy by taking into account individual behavior. On the other hand, compared to covariates, they generally do require a calibration phase, i.e., training a classification algorithm. As for behavioral metrics interesting for prolonged use, to monitor mental fatigue in drivers, steering (McDonald et al., 2012; Li et al., 2017) and the combination of multiple measures (Wakita et al., 2006) have proven effective. Pimenta et al. (2013) tested a multitude of metrics extracted from mouse and keyboard behavior during a task to detect mental fatigue. Six out of 15 Metrics were capable of classifying fatigue. These included keydown time, time between keys, mouse acceleration, mouse velocity, time between clicks, and errors per key. As with the covariate metrics and the study combining several driving metrics, Pimenta et al. (2013) also confirmed the superior performance of using a combination of several metrics over a single one.

### 4.3. Physiological metrics

Research in physiological computing -and passive brain-computer interfaces when one cerebral metric is used for estimation. Roy et al. (2020) have identified several task independent metrics that can be used with high precision. The downsides are that training data need to be acquired, as well as the intrusiveness of many systems, making them less practical. Regarding cerebral metrics, increases in EEG power of the alpha (8-12 Hz) and the theta (4–7 Hz) bands have been repeatedly associated with increased mental fatigue (Borghini et al., 2014; Roy et al., 2014; Jahanpour et al., 2020; Tran et al., 2020). EEG based connectivity measures have also been successfully used to predict performance in a simulated driving task (Sun et al., 2014). Park et al. (2021) proved that mobile setups can also function during more ecologically oriented tasks such as UAS operations. In an actual flight condition, Dehais et al. (2018) classified cognitive fatigue based on functional near infra-red spectroscopy (fNIRS; blood oxygenation measurement) and EEG connectivity measures. Ahn et al. (2016) showed that mental fatigue resulted in increases in frontal oxygenation.

Electrooculography (EOG) and the eye-tracking study also had success in detecting fatigue. Considerable discourse exists in the literature as to what metrics are most useful for detecting the effects of prolonged continuous use, as eye movements may differ substantially depending on the task. In a review of aviation and automobile studies, Borghini et al. (2014) found that TOT resulted in decreases in both blink duration and blink rate. Another metric shown to have a strong association with TOT in both naturalistic as well as highly controlled experiments is the saccadic velocity (Di Stasi et al., 2013). Roy et al. (2016a) studied the effects of prolonged continuous use during Unmanned Aerial System UAS operations and found that fixations, as well as focus on areas of interest, significantly decreased as TOT increased.

The last measure that is consistently used for TOT estimation is cardiac activity. Research indicates that TOT results in decreases in heart rate (HR) (Lal and Craig, 2002; Ahn et al., 2016) and increases in heart rate variability (HRV), the variance between heart beats (Zhao et al., 2012; Borghini et al., 2014; Ahn et al., 2016; Melo et al., 2017; Huang et al., 2018). As with the other measures, the combination of several metrics also seems to result in improved estimation (Ahn et al., 2016). Beyond these transitional measures, the researchers have also found that posture can determine TOT and mental fatigue during driving simulations (Furogori et al., 2005; Ansari et al., 2021). Finally, the merging of multiple metrics into useful measures for their specific application has also seen success. This was done by Azim et al. (2014) for drivers and by Li et al. (2020) for construction equipment operators.

## 5. Discussion

The goal of this article was to explore the potential of adaptive systems, and mental state-based adaptive interfaces in particular, for use during prolonged continuous operations. The literature shows that with increasing TOT, cognitive flexibility, attention, and situational awareness decrease, resulting in a higher risk of error and failure during the operation of complex tasks (Blackmon and Gramopadhye, 1995; Bliss et al., 1995; van der Linden et al., 2003a,b; Lorist et al., 2009; Holtzer et al., 2010; Csathó et al., 2012; Krishnan et al., 2014; Sun et al., 2014; Plukaard et al., 2015; Guo et al., 2016; Petruo et al., 2018; Wickens and McCarley, 2019; Yu et al., 2021). The effectiveness of adaptive interfaces, built to mitigate said effects is determined by a multitude of factors: Operators need to understand the system they are working with (Scott et al., 2006; Kiefer et al., 2017; Xing et al., 2021) and also trust the system (Lee and See, 2004). Furthermore, ecological validity needs to be accounted for in order to avoid problems with secondary effects (Chen and Barnes, 2014), boundary conditions (Pritchett et al., 2014), scalability (Hou et al., 2007), calibration (Taylor et al., 2013), and complexity (Lajos, 1995; Höök, 2000; Hou et al., 2007). The adaptations themselves may take many forms, ranging from highlighting targets (Parasuraman et al., 2009), to attentional alerts (Taylor et al., 2013; Saint-Lot et al., 2020) and information removal (Peysakhovich et al., 2018). Regarding the detection of a degraded mental state due to prolonged use, it was shown that a combination of measures, with the addition of a reliable estimation method, outperforms single measures (Wakita et al., 2006; Pimenta et al., 2013; Ahn et al., 2016; Sikander and Anwar, 2019). Furthermore, studies combining several entities such as behavioral and physiological measures reduce the disadvantages of single metrics and may also provide redundancy of measures (Azim et al., 2014; Li et al., 2020). Further details on these findings are summarized in Tables 1–3.

When looking at the body of literature reviewed for this article, it becomes evident that to this date relatively few complete mental state based adaptive interfaces have been developed and tested (Piechulla et al., 2003; Parasuraman et al., 2009; Aricò et al., 2016; Park et al., 2021). In the future, more complete systems should be developed and tested as only complete systems allow for the evaluation of certain factors such as trust (Lee and See, 2004). If the goal of these interfaces is to be incorporated into the daily complex operations that happen around the world, then researchers and designers should at all times ask themselves whether the system that they are working on could actually be applied in a real operation. But also here practical limitations have to be considered. Machine learning algorithms can still require long calibration phases depending on the metric that is being used (Aricò et al., 2017), and certification into use may be a lengthy process depending on the domain of the operations (Torens et al., 2021). The question of trust in adaptive interfaces has, to the best of our knowledge, not been explored yet, but may prove to be a further component to be considered in future research.

This article is the first step toward better understanding what is known, what currently exists, and what remains to be achieved regarding prolonged use in human-machine interaction and solutions such as adaptive interfaces. Yet, some aspects were voluntarily put aside and remain to be investigated. Among those one can list the interaction between prolonged use and sleep cycles, circadian rhythms, and shift work.

The relation between sleep and mental fatigue is complex and difficult to explore, as definitions and experimental procedures vary widely (Åkerstedt et al., 2004; Matthews, 2012; Jahanpour et al., 2020). The impact of prolonged use depending on solicited sensory modality is also to be studied, as well as the interaction with individual differences of operators, such as expertise and preferences.

It also needs to be acknowledged, that the effects of mental fatigue on cognition that are discussed in this review do not form an exhaustive list of aspects in which mental fatigue may alter cognition. Finally, a topic closely linked to prolonged use is that of learning. Indeed, learning, at least in the -possibly virtual- classroom context presupposes spending an extended amount of time on exercises and complex tasks, with potential repetitions. A few authors have started investigating adaptive solutions for e-learning and digital reading making use of users' mental state estimations with success (Walter et al., 2017; Andreessen et al., 2021). Yet, while it may benefit from adaptive interfaces, to our knowledge the interaction between learning and prolonged use of dedicated interfaces has not been assessed. A further limitation of the body of work presented here can be seen in the different TOT that was used in different studies to elicit mental fatigue Table 1. Different durations ranging from 35 to 180 min need to be taken into account when comparing the outcomes of studies. To the best of our knowledge, there is no general consensus on when mental fatigue occurs as this likely depends on the task, individual, and other factors (Matthews, 2012). However, by testing different rest periods between blocks of demanding tasks, Lim et al. (2016) present an interesting framework for deciding how long a task should be performed. A further limitation of the here presented body of work is that machine learning and related aspects such as transfer learning and practical aspects of brain-computer interfacing were left undiscussed (Brouwer et al., 2015; Zeng et al., 2021). For instance, TOT has been shown to significantly impact the distribution of classical features for mental workload estimation, as well as the accuracy of pipelines that relied on these measurements (Roy et al., 2013, 2016b).

## 6. Conclusion

**Figure 2:**
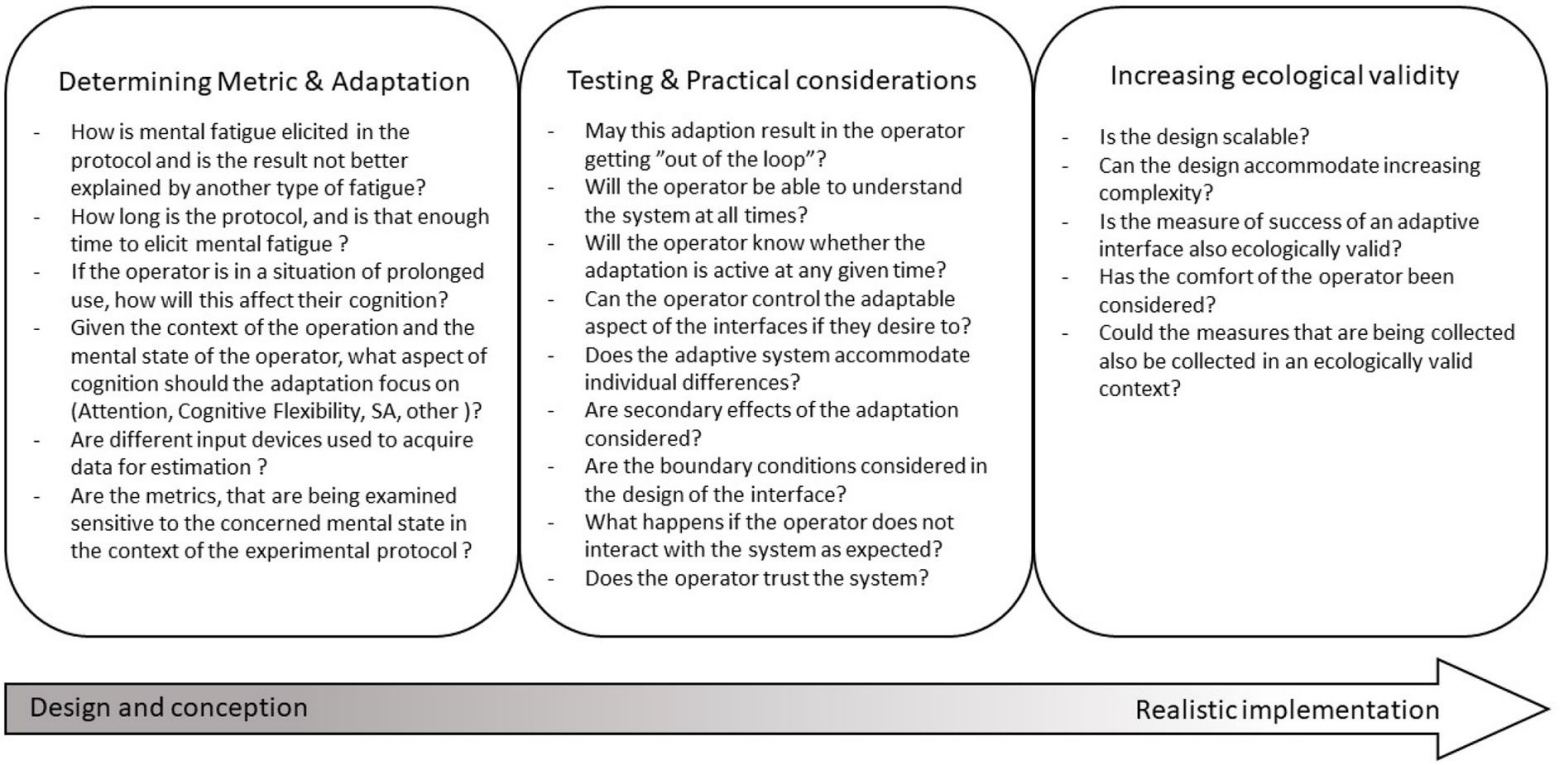
Proposed, non exhaustive set of questions, for consideration during adaptive interface conception design and testing.

Advances in technology as well as the trend toward ever more complex systems will likely increase the demand for more sophisticated and also more complex adaptive interfaces, in order to counteract the effects of prolonged continuous use. The incorporation of user and operator mental state estimations may in the future prove to be one of the pivoting factors for their success (Szostak, 2013). Mental state-based adaptive interface design incorporates research from cognitive science, neuroergonomics, human factors, and human-machine interaction as well as the research and particular characteristics of each domain of application (e.g., aviation and automobile). The necessity for researchers from one domain to gain proficiency in other domains due to interdisciplinarity was a driving factor for this article. To maximize the success of future mental state based adaptive interfaces, researchers and designers should take the points summarized in Figure 2 into consideration. These questions form the conclusions that can be drawn from this article and facing them before designing, implementing and testing a mental state based adaptive interface may help during the process and may increase the relevance and efficiency of a design. In the future, systems, as are reviewed and proposed here, will hopefully become more relevant, allowing for increased safety during complex operations.

## Author contributions

MH, RR, and AB: drafting of the article. RR and AB: supervision. All authors contributed to the article and approved the submitted version.

## Funding

This study was funded by the French Defence procurement and technology agency (DGA) as part of the publicly funded Concorde Project.

## Conflict of interest

The authors declare that the research was conducted in the absence of any commercial or financial relationships that could be construed as a potential conflict of interest.

## Publisher's note

All claims expressed in this article are solely those of the authors and do not necessarily represent those of their affiliated organizations, or those of the publisher, the editors and the reviewers. Any product that may be evaluated in this article, or claim that may be made by its manufacturer, is not guaranteed or endorsed by the publisher.
